# Characterising the killing of girls and women in urban settings in Latin America, 2000–2019: an analysis of variability and time trends using mortality data from vital registration systems

**DOI:** 10.1136/bmjph-2024-000985

**Published:** 2024-07-24

**Authors:** Bricia Gonzalez Trejo, Yvonne L Michael, Ana V Diez Roux, Brisa N Sánchez, Nina Sun, Heidi Stöckl, Dèsirée Vidaña-Perez, Catalina Correa-Salazar, Ana Ortigoza, Amélia Augusta de Lima Friche, Vanessa DiCecco, Mónica Mazariegos, Usama Bilal

**Affiliations:** 1 Department of Epidemiology and Biostatistics, Drexel University Dornsife School of Public Health, Philadelphia, Pennsylvania, USA; 2 Drexel University Urban Health Collaborative, Philadelphia, Pennsylvania, USA; 3 Article XII Consulting, Chapel Hill, North Carolina, USA; 4 Institute for Medical Information Processing, Biometry and Epidemiology, Ludwig-Maximilians-Universität München, Munich, Germany; 5 Pettenkofer School of Public Health, Munich, Germany; 6 Department of Health Promotion, Education, and Behavior, University of South Carolina Arnold School of Public Health, Columbia, South Carolina, USA; 7 Departamento de Psicología, Universidad de los Andes, Bogota, Colombia; 8 Department of Social and Environmental Determinants for Health Equity, Pan American Health Organization, Washington, District of Columbia, USA; 9 Universidade Federal de Minas Gerais, Belo Horizonte, MG, Brazil; 10 Universidad Nacional de Lanus, Lanus, Buenos Aires, Argentina; 11 INCAP Research Center for the Prevention of Chronic Diseases (CIIPEC), Institute of Nutrition of Central America and Panama (INCAP), Guatemala, Guatemala, Guatemala

**Keywords:** Epidemiology, Public Health, Female, trends

## Abstract

**ABSTRACT:**

**Introduction:**

Latin America is burdened by high levels of violence. Although boys and men often experience more violence and fatalities, girls and women face a greater risk of being killed by family members or intimate partners due to their gender, a phenomenon known as femicide. Our study estimates femicide rates in Latin America across age groups, examining city-level variations and temporal trends.

**Methods:**

Utilising data from the *Salud Urbana en America Latina* project, we analysed mortality data from 343 cities in nine countries between 2000 and 2019. We calculate the variability between and within countries using data from 2015 to 2019. We then describe time trends using femicide counts by year and city and fitting a three-level negative binomial model with a random intercept for country, fixed effects for age categories, and city-level and country-level random slopes for time (scaled to decades). Finally, we assess longitudinal time trends by age by including an interaction term for age and time (scaled to decades).

**Results:**

Our results highlight substantial heterogeneity in femicide rates within and between countries. Additionally, we find that women 15–29 and 30–44 years of age experience the highest femicide rates across all countries. While our findings suggest a slight decline in femicide rates per additional decade (RR 0.95, 95% CI: 0.74 to 1.24) between 2000 and 2019, the trends diverge in different countries, suggesting increasing rates in some countries like Mexico. Age-specific trends suggest the persistence of higher rates among women 15–29 and 30–44 years of age over time.

**Conclusion:**

We underscore the need to consider gender dynamics in understanding and preventing femicides, focusing on city-level interventions to address the multifaceted causes of violence against girls and women in the region.

**WHAT IS ALREADY KNOWN ON THIS TOPIC**

- Latin America is home to 18 of the 25 countries with the highest femicide rate. Some region-specific evidence of within country heterogeneity exists, but no studies have examined femicides in Latin America at the city level over time or how these trends vary by age.

**WHAT THIS STUDY ADDS**

- The substantial within country heterogeneity suggests that femicides may be in part conditioned by social structural characteristics of the cities. Data from the last two decades suggest divergent country trends and hint at rising rates in Mexico and stagnation elsewhere. Moreover, trends show sustained and higher rates in women 15–29 and 30–44 years of age.

**HOW THIS STUDY MIGHT AFFECT RESEARCH, PRACTICE OR POLICY**

- Generating data on femicides is a key strategy for shaping policy priorities for prevention. Previous comparative studies have used country-level estimates to produce time trends analysis or focus on describing differences between males and females. Such estimates may mask within country and within group variability in femicide rates. Future research should explore the city-level structures that may be related to femicide trends.

## Introduction

 Latin America is one of the most violent regions in the world, home to 18 of the 20 countries with the highest homicide rates worldwide.^1^ Violence against girls and women is a major public health problem in Latin America where, depending on the region, an average of 21%–38% of women 15–49 years of age report suffering physical or sexual violence by men in their lifetime.^2^ Although boys and men are often more likely to experience violence and to die from it, girls and women are more likely to be killed by family members or intimate partners and more likely to be killed because of their gender, a phenomenon known as femicide.^3^

Yet, studies using a gendered perspective on homicide tends to be reduced to sex-stratified studies that describe between-sex differences.^4^ These comparisons are often a necessary step towards understanding health inequities considering data limitations, but they are not sufficient.^5^ By ignoring the gender dimension of female homicide, these studies obscure the persistence of the problem and fail to address power dynamics. It is precisely the socially constructed power imbalance between girls/women and boys/men, exacerbated by the patriarchal belief that men and boys can and should express themselves through violence, that drives most female homicides.^6^ Hereafter, we will use the term *femicide* to describe this phenomenon. We acknowledge that our data, like most, reflect sex-specific estimates, but we nonetheless use gender terms like girls and women and the term femicide to stress the importance of exploring female-specific homicide rates through a gendered lens.

Nearly all countries in Latin America have enacted legislation against femicide either as part of their penal code or as standalone legislation.^7^ Yet, femicide remains a contested legal category across the region, and how it is treated inside the legal system has important implications for its measurement. While some countries limit their definition to intimate partner relationships, others broaden the definition but require evidence of motive, sexual violence, and/or previous instances of violence.^7^ Moreover, some countries in the region use the term feminicide, a term introduced by Mexican scholar Marcela Lagarde, to emphasise the gender-based power dynamics embedded in the murder of girls and women and the state’s complicity in these crimes.^8^ Researchers and activists alike have pointed to the fact that these varying definitions often do not allow for cross country or even within country comparisons of gender-related killings of girls and women.^9^,^11^ Yet, beyond the difficulty encountered in comparative research, relying on legal definitions is problematic because of the many victims that are left out of legal frameworks across the region. Decisions about which femicides are included or excluded from a country’s legislation are linked to the historic and social contexts of each country. It is important to note that this problem is not exclusive to femicides. All indicators are human constructs and in fact, the definition of the less contested and more widely used homicide indicator also varies by region.^12^

Globally, 89 000 women and girls were killed intentionally in 2022.^13^ Evidence suggests that globally femicide trends have remained stable.^13 14^ While recent studies highlight the great variability in risk and trends between and within countries,^13^,^22^ some similar trends across countries have emerged over the years. Studies have found that femicide rates are highest among young adult women,^1920 22^,^24^ individual-level factors including lower education and lower income increase the risk of femicide^25 26^ and current legislation remains limited in its ability to identify and prevent femicides.^27 28^

Studies that have explored the Latin American region have found large heterogeneity in femicides between^13 20^ and within countries,^16^,^1921 22^ mostly at the subnational level. However, Latin America is one of the most urbanised regions worldwide^29^ and previous research has shown that urban areas tend to have higher levels of violence^30^ and within urban areas, larger cities tend to have higher violence overall,^31 32^ suggesting that national and subnational estimates of femicide may mask high city rates. Moreover, evidence from the region suggests that in many countries femicide rates have either remained stable or increased over time.^14 20 22^ One study which analysed femicides across all ages in five Latin American countries between 2001 and 2011 found that the average femicide rate between 2001 and 2011 remained stable in Chile and increased over time in Brazil and Mexico.^20^ A more recent, country-specific, study in Brazil found that femicides among women 15–49 years of age have remained stable between 1990 and 2019, highlighting the importance of exploring age specific trends.^22^

Prior research in Latin America indicates that there are considerable differences in femicide rates and trends across age groups. Most studies examining femicide across age groups suggest higher rates in adolescents and young adult women^19 20 22 24^ but age-specific trends over time have varied across the region. One study found that in Mexico, femicide rates in those 15–29 years of age rose by nearly 9% on average in the decade between 2001 and 2011. This is higher than an overall change of 5.7% across the studied countries which also included Argentina, Brazil, Chile and Colombia.^20^ In contrast, in Brazil, between 2001 and 2011, the highest average annual increase (8% with 95% CI: 5.15% to 12.55%) was seen in younger girls 0–14 years of age.^20^

Despite national-level and regional-level evidence that femicide rates are highest in urban areas, and available evidence of heterogeneity across age group and time, few research studies have examined femicide trends more recently at the subnational level. Moreover, recent empirical evidence on the city-level distribution and trends of femicides by demographic characteristics remains rare, despite the 2030 Sustainable Development Goals of understanding and preventing within-country inequities as well as preventing gender-based violence such as femicide.^33^ The lack of comparative research is likely due to the difficulty in reconciling what counts as femicide across countries. This study builds on previous results by using mortality data from vital registration systems to proxy femicides at the city level over two decades and exploring within-group variation by age.

We use harmonised homicide data from 343 cities with 100 000 residents or more in nine Latin American countries to: (1) estimate femicide rates and quantify variability between and within countries, (2) describe age-specific femicide rates and (3) quantify changes in femicide rates in these cities over time and by age. Given the available evidence, we hypothesise that there will be greater variability in femicide rates within countries than between countries. We also hypothesise that mortality will be greatest in adolescent and young adult women and that femicide trends have increased with time specifically in younger women.

## Methods

### Data

Mortality and population data were obtained from the *Salud Urbana en America Latina* project (SALURBAL), a multicountry project exploring determinants of urban health in 371 cities across 11 Latin American countries including Argentina, Brazil, Chile, Colombia, Costa Rica, El Salvador, Guatemala, Nicaragua, Mexico, Panama and Peru. These 371 cities constitute the entire universe of cities with a population >100 000 in these 11 countries^34^ and are the result of an aggregation of local administrative areas (eg, *municipios*) that cover the urban extent of each city as determined by satellite imagery.^34^ In this study, we include all cities and countries with multiple years of mortality data between 2000 and 2019, resulting in 343 cities from nine countries: Argentina, Brazil, Chile, Colombia, Costa Rica, El Salvador, Guatemala, Mexico and Panama. Table A (available as an online supplemental file) shows the years of data available for each country. When a country had data for any given year, this meant that all cities within the country had data for that year. The average number of years across countries was 18.1 (SD 3.1). Brazil, Chile, Colombia, Costa Rica and Mexico had data for all 20 years and Guatemala and Argentina both had the lowest number of years of data available with 11 and 15 years of data each.

Mortality data were obtained from vital registration systems in each country for years available between 2000 and 2019. Details on how mortality data were processed are detailed elsewhere^34^. Briefly, deaths were categorised using the Global Health Estimates (GHE) codes to aggregate the International Classification of Diseases 10th version (ICD-10) into larger groupings of causes of death. We also accounted for ill-defined deaths by redistributing them to other categories, and for the undercounting of death counts using methods described elsewhere.^32 34^ As denominators, we obtained population projections and intercensal population estimates from national census bureaus in each country.

### Outcome

We used female homicides as a proxy for femicides. The measurement of femicide is a complex process and influenced by divergent strategies.^10 11^ A more nuanced exploration of the different ways femicides and the related term feminicides have been operationalised globally and in Latin America and the important implications of each decision has been explored elsewhere.^79^,^11 35^ An important unresolved debate is whether a female victim is sufficient to identify a killing as femicide.^11^ Some researchers point out that while the proxy may be less nuanced, considering well-documented difficulties in establishing motive and the lack of consensus on gender-based indicators, the use of homicide data that capture the killing of women by men is the most appropriate for cross-country comparisons as vital registration data are less influenced by legal decisions.^27^ Yet, femicide can be perpetrated by any gender even if it is more often committed by boys and men.

Our decision to use female homicide as a proxy for femicide, however, stems from the ideas of other feminist theorist who have argued that the pervasiveness of gender inequality and impunity in the region itself generates the conditions for the killing of women.^35 36 39 40^ Although in theory, femicides are a specific phenomenon within female homicides, given the evidence of gender inequality and violence against girls and women in the region, we situate our work within the theoretical framework that all killings of feminine bodies in a context of rampant gender inequality serve as a good proxy for femicide. We argue that specific indicators driven by legal frameworks limit our understanding of the problem and deepen power inequalities in the region by failing to acknowledge the true extent of the marginalisation of girls and women. Moreover, countries where femicide is only considered under domestic relationships amounts to a ‘degendering’ of the issue.^41^ To more accurately and consistently recognise femicide, we must centre what Fitz-Gibbon and Walklate^35^ call ‘ordinary violence’ which are embedded within social structures. While less nuanced administrative data presents its own challenges, we argue that it is a good starting point for capturing the true extent of the violence experienced by girls and women in the region. Moreover, our analytic choice here is consistent with several prior studies.^14 16 42^

Specifically in our study, we identified homicide specific deaths using GHE code 1580 for violence (ICD-10 codes X85-Y09 and Y87.1), exclusively for decedents identified as female. We categorised femicides in the following age groups: 0–14, 15–29, 30–44, 45–59 and 60+ years of age which are distributions used in the one other study that focused on multicountry exploration of femicide trends in this region^20^ and because they represent important life periods previously used by the United Nations Economic Commission for Latin America and the Caribbean (ECLAC) which publishes statistics covering the region.^43^ For the longitudinal analysis, femicide counts were aggregated at the city level for each of the same age groups.

### Statistical analysis

We conducted this analysis in four steps. First, we pooled data between 2015 and 2019, the most recent 5 year period for which we have data for all countries. We then calculated age-specific rates per 100 000 female inhabitants for each city. We then calculated age-standardised rates using the WHO 2000–2025 World standard population.^44^ To describe the heterogeneity of femicide between and within counties, we computed the intraclass correlation coefficient (ICC) using an empty two-level linear model (cities within countries) with the log age-adjusted femicide rate as the outcome. For the purposes of calculating the ICC, we excluded the one city that had a rate of zero (the city of Chillan in Chile). Details on the number of cities and city-years reporting zero femicides can be found in table B (available as an online supplemental file). To visualise city-level femicide data, we mapped femicide rates per 100 000 females using quartiles as categories. Second, we assessed age-specific femicide rates by city across all countries using the same pooled data between 2015 and 2019.

Third, we examined longitudinal time trends from 2000 to 2019 in age-adjusted femicide rates by city and country using spaghetti plots to identify changes in slope. We describe time trends using femicide counts by year and city and fitting a three-level negative binomial model described below with a random intercept for country, fixed effects for age categories and fixed and city-level and country-level random slopes for time (scaled to decades), along with an offset for population:

$$\begin{array}{ll} \log\left(Femicide_{ijk}\right)= & \alpha_{000}+U_{00k}+U_{0jk}+\left(\left(\alpha_{100}\right)+\left(U_{10k}\right)+\left(U_{1jk}\right)\right)\\ T_{ijk}+\alpha_{200}\left(Age0-14\right)+\;\alpha_{300}\left(Age30-44\right)+\\ \alpha_{400}\left(Age45-59\right)+\;\alpha_{500}\left(Age60+\right)+log\left(pop_{ijk}\right) \end{array}$$

In this model, Femicide_*ijk*_ is measurement at time *i* on city *j* in country *k*. *T*_ijk_ is year (scaled to decade) for measurement *i* on city *j* in country *k*. We rescaled time to decades to facilitate interpretation of trends as 10 year changes. Furthermore, $\alpha_{000}$ = is the intercept measuring the overall femicide rate, $\mu_{00k}$ is a random intercept for country *k* and $\mu_{0jk}$ is a random intercept for city *j* in country *k* and the overall slope is given by $\text{(}\alpha_{100}\text{)}T_{ijk}$, the country-specific slope is $\left(\alpha_{100}+U_{10k}\right)\;T_{ijk}$, the city specific slope is given by $\left(\alpha_{100}+U_{10k}+U_{1jk}\right)T_{ijk}$ and $log\left(pop_{ijk}\right)$ is the offset variable for the log transformed population. Equation A (available as an online supplemental file) shows the model equation by level. Last, to assess longitudinal time trends by age, we include an interaction term for age and time (scaled to decades).

All analyses are conducted using R statistical software (R V.4.2.1).

## Results

In the 343 included cities in nine countries, 116 133 femicides were reported and registered among all ages between 2000 and 2019 (inclusive of different time periods for the different countries) and 32 655 femicides were reported and registered between 2015 and 2019.

### Variability and rate of femicide in Latin American cities

**Figure 1:**
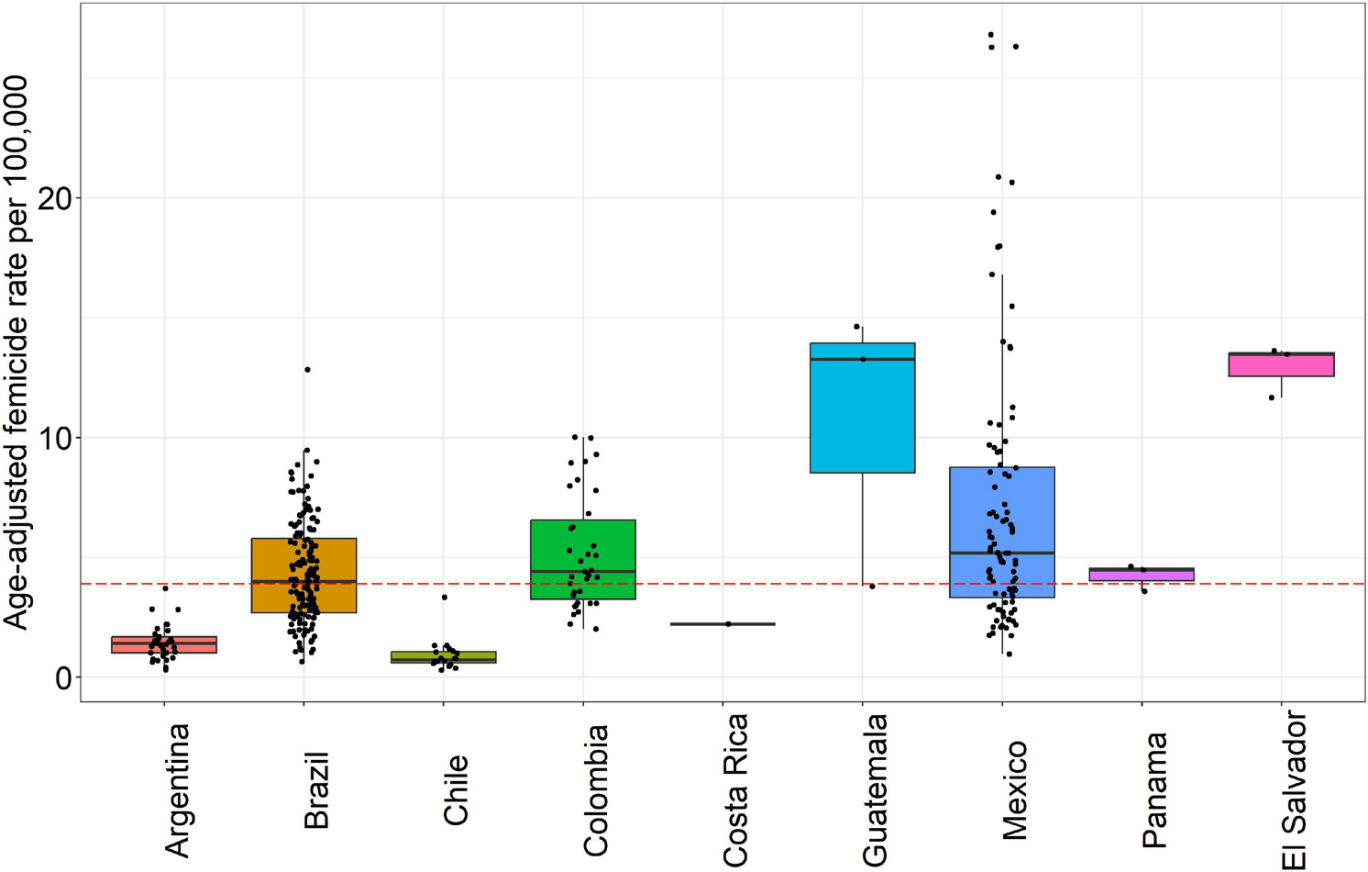
Distribution of femicide rates in cities by country, 2015–2019 (n=343 cities). Each dot represents a city-estimate of femicide rate, and boxplots show the country distribution of femicide rates. Dashed red line describes the median city-level femicide rate of the overall sample (3.9 per 100,000 females). Only the city of San Jose is included for Costa Rica, so the line corresponds to femicide rate in that city.

The overall median city femicide rate between 2015 and 2019 was 3.9 per 100 000 females (IQR=2.4–6.1) across the 343 cities (figure 1). Argentina, Chile and Costa Rica all had lower median city femicide rates while Brazil, Colombia, Guatemala, Mexico, El Salvador and Panama all had higher median city femicide rates. Although the femicide rates differed across cities within countries, the greatest heterogeneity is observed between countries. The ICC estimation showed that 67% of the variability occurs between countries and 33% between cities within countries.

**Figure 2:**
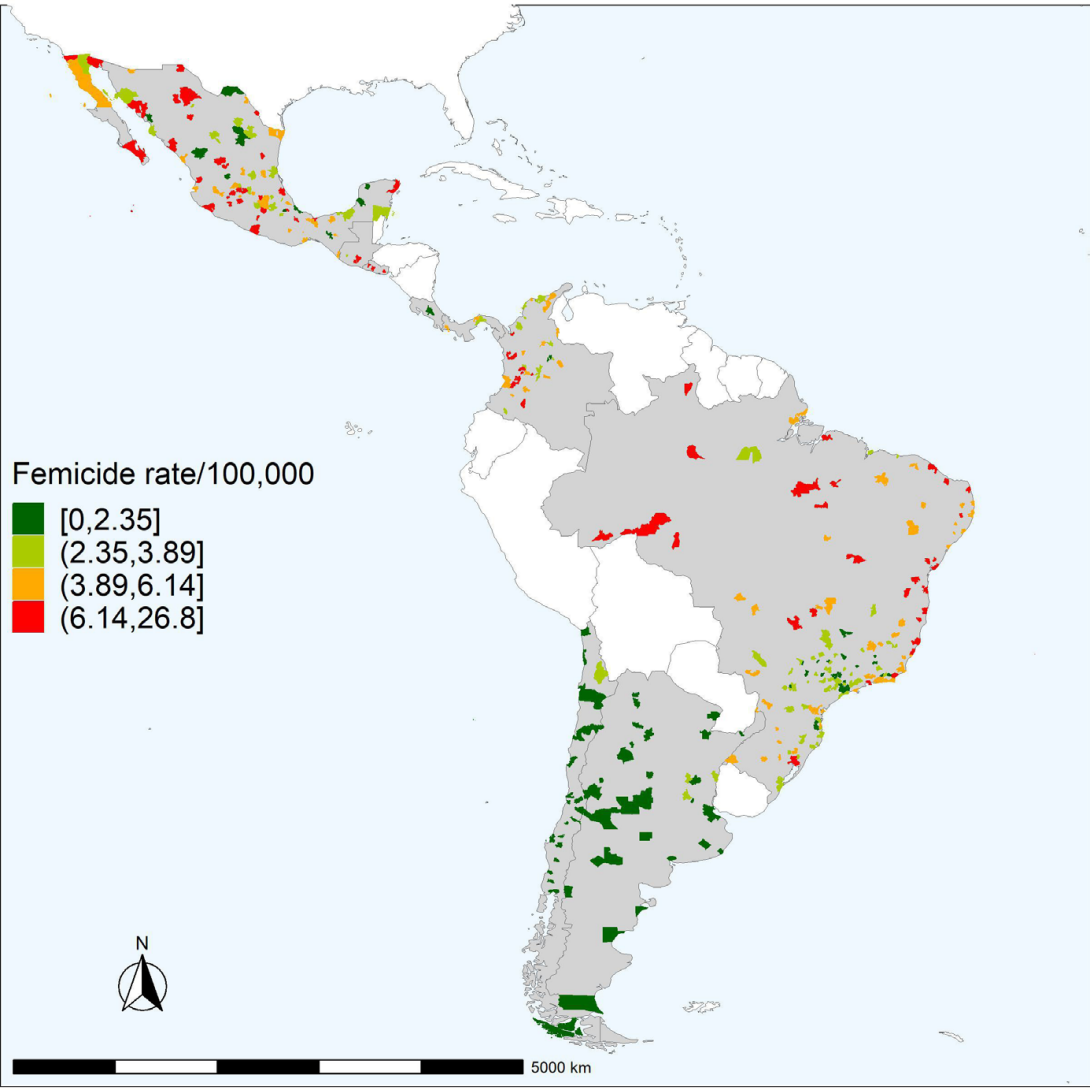
Spatial distribution of age adjusted femicide rate by city in 343 Latin American cities 2015–2019.

Figure 2 shows the spatial distribution of the city-level age adjusted femicide rates within countries for the 2015–2019 period. In Mexico, Acapulco in the state of Guerrero, on the southwestern coast had the highest reported femicide rate in the region, with a rate of 26.8 per 100 000 females, approximately five times Mexico’s median city-level femicide rate of 5.2 100 000 females. The lowest femicide rate is reported in Chile. Chillan in the region of Nuble, Chile reported no femicides between 2015 and 2019. There are clear spatial patterns within countries. For example, in Brazil, femicides were highest in the North and Northeast, and lowest in the Southeast. In Colombia, femicides are highest along the Western and central regions. In Mexico, the highest femicide rates are along the Mexico-US border and along the Pacific coast.

### Femicides by age group

Overall, the highest median city femicide rate is seen for women 15–29 years of age, while the lowest is seen for girls 0–14 years of age (6.12 and 0.90 per 100 000 females, respectively) (table 1). Across most countries, the age-specific rates were also highest for women 15–29 years of age. Guatemala showed the highest median city femicide rate for women 15–29 years of age at 29.08 per 100 000 females, over four times the overall median city femicide rate in this age group. Other countries including Brazil, Colombia, Mexico and El Salvador also had median city femicide rates above the overall median for this age group. While the highest city femicide rate in Argentina, Chile and Costa Rica is also among women 15–29 years of age, the median in these countries is lower than the overall median city femicide rate. El Salvador and Panama exhibited higher median city femicide rates for women 30–44 years of age. For Guatemala the difference between women 15–29 years of age and 30–44 years of age was greatest (14.14 vs 19.08 per 100 000 female inhabitants, respectively). El Salvador reports some of the highest median city femicide rate across all age categories and there is relatively little difference among women over 15 years of age.

**Table 1:** Median (Q1, Q3) city femicide rates per 100 000 within Latin American countries from pooled analysis 2015–2019

	Age category				
	0–14	15–29	30–44	45–59	60+
Overall	0.90 (0; 1.54)	6.12 (3.09; 10.50)	5.67 (3.26; 9.06)	3.27 (1.66; 5.03)	2.52 (1.36; 4.49)
AR	0.34 (0; 0.61)	1.97 (1.29; 2.62)	1.76 (1.26; 2.60)	1.24 (0.90; 2.02)	1.64 (1.11; 2.32)
BR	1.01 (0; 1.57)	6.38 (3.54; 9.89)	6.31 (4.18; 8.35)	3.32 (1.88; 4.53)	2.40 (1.39; 3.62)
CL	0 (0; 0.21)	1.08 (0; 1.18)	1.07 (0; 1.22)	0.98 (0; 1.69)	0.62 (0; 1.27)
CO	1.08 (0.32; 1.75)	7.61 (5.18; 10.03)	5.86 (4.63; 11.26)	3.69 (2.75; 5.05)	2.77 (1.87; 3.55)
CR*	0.65	4.44	2.66	1.59	1.25
GT	0.93 (0.91; 1.93)	29.08 (17.30; 29.22)	14.14 (9.63; 19.80)	4.38 (3.18; 6.83)	5.78 (4.49; 9.05)
MX	1.08 (0.60; 1.64)	8.8 (5.11; 14.43)	7.44 (3.86; 13.64)	4.9 (2.78; 7.34)	4.44 (2.40; 6.31)
PA	2.04 (1.66; 2.10)	5.42 (5.36; 6.18)	9.13 (6.28; 9.66)	1.81 (1.48; 2.64)	3.9 (1.95; 4.16)
SV	1.30 (1.24; 1.87)	15.57 (15.02; 18.36)	18.69 (17.3; 20.05)	12.6 (12.54; 13.88)	12.47 (11.29; 25.44)

* Only the city of San Jose is included for Costa Rica, so the age -specific femicide rate corresponds to rate in that city.

AR, Argentina; BR, Brazil; CL, Chile; CO, Colombia; CR, Costa Rica; GT, Guatemala; MX, Mexico; PA, Panama; SV, El Salvador

### Time trends

**Figure 3:**
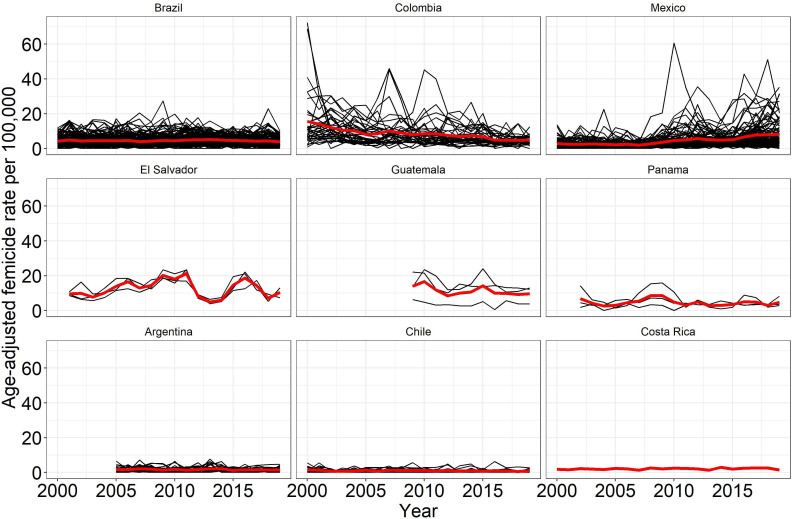
Spaghetti plots by country showing age adjusted femicide rates per 100 000 females for different cities (black lines) across time and showing average country-level trend (red line). Only the city of San Jose is included for Costa Rica, so the red line corresponds to trends in that city.

The time trends in city femicide rates across countries are shown in figure 3. The country with the highest average city (age-adjusted) femicide rate between 2000 and 2019 was El Salvador (12.6 per 100 000 females) and the country with the lowest average city femicide rate was Chile (1.0 per 100 000). Overall, cities in Argentina, Chile and Costa Rica have lower femicide rates during this period than the other countries. Cities in these three countries show little change over time. Similarly, Brazil, a country with higher average city femicide rates than Argentina, Chile and Costa Rica, on average shows little change over time. Meanwhile, Colombian cities exhibit a downward trend between 2000 and 2019, and Mexican cities appear to trend upward during the same period. Cities in Central American countries show greater variability. El Salvador maintained an upward average city femicide trend until 2011 when femicide rates declined drastically and then increased again in 2015.

Certain rises in city femicide rates within countries are evident across all countries. In Brazil, for example, the highest peak in femicide was seen in 2009 in the city of Linhares (27.3 per 100 000 females) and Porto Seguro in 2018 (23 per 100 000 females). In Colombia, the highest femicide rates were reported in the year 2000 in the city of Florencia situated in the department of Caqueta (72.2 per 100 000 females), and Apartado in the department of Antioquia (69 per 100 000 females). Apartado peaks again in 2007 after a period of slow decline (46 per 100 000 females). The second peak in 2010 refers to the port city of Buenaventura in the department of Valle de Cauca (45.2 per 100 000 females). In Mexico, the largest peak was seen in Ciudad Juarez in 2010 (61 per 100 000 females). In Panama, Colon, the capital city of the department of Colon exhibited the highest rates in 2002 and peaks again in 2009 (14.3 per 100 000 and 16 per 100 000 females, respectively).

Table 2 presents the exponentiated incidence rate ratios of femicide associated with time in decades (model 1) and after adjusting for age (model 2). Results from model 2 (the age-adjusted model) suggest that the femicide rate declines by 5% per decade, although there was not enough evidence to rule out the null hypothesis of no changes in femicide rates over time (95% CI: 0.74 to 1.24). However, we found variability between countries in these time trends. For Mexico, random-effect estimates from our model $U_{00k}$, or the deviation to the median baseline values; and $\left(\alpha_{000}\text{+}U_{10k}\right)$,or the deviation to the median trend of 5% decline, suggest a positive slope for femicide rates over time, different from the average slope over all countries. Figure A (available as an online supplemental file) illustrates between country variance, showing country specific intercepts and slopes from model 2 or the country-specific risk of femicide as well as country-specific changes over time and how these differ from overall averages. These country-specific estimates suggest that particularly in Mexico femicide rate increases over time between 2000 and 2019. The great variability in country-specific intercepts and slopes also confirms the strong effect of country on femicide mortality.

**Table 2:** Longitudinal analysis of femicides in Latin American cities 2000–2019: incidence rate ratios (RR) with 95% CI for different age groups and over time

	Model 1	Model 2*	Model 3*
	RR (95% CI)	RR (95% CI)	
Time (decades)	0.96 (0.75 to 1.24)	0.95 (0.74 to 1.24)	
Age (0–14 years)		0.17 (0.16 to 0.17)	
Age (15–29 years)		1 (Ref.)	
Age (30–44 years)		0.86 (0.84 to 0.88)	
Age (45–59 years)		0.54 (0.53 to 0.56)	
Age (60+ years)		0.48 (0.47 to 0.50)	
Time (in age 0–14 years)			**0.81 (0.77 to0.86**)
Time (in age 15–29 years)			1.02 (0.79 to 1.31)
Time (in age 30–44 years)			0.98 (0.94 to 1.02)
Time (in age 45–59 years)			**0.89 (0.85 to0.93**)
Time (in age 60+ years)			**0.80 (0.76 to0.85**)

Countries include Argentina, Brazil, Chile, Colombia, Costa Rica, El Salvador, Guatemala, Mexico, and Panama.

Bold values indicate statistically significant estimates at a p<0.05.

* Results for model 2 show the time trend, overall, while adjusting for age; rResults for model 3 show linear combination of the main effect and interactions coefficients, representing the time trend (scaled to decades) for each age group.

Our final model presents predicted trends in femicides by age group (model 3) which includes an interaction for age and time. Results suggest that for every additional decade, the average femicide rate for women 15–29 increases by 2% (95% CI 0.79 to 1.31), although again, there was not enough evidence to rule out the null hypothesis of no change in femicide rates. Results for all other age groups suggest a decline. Except for women 30–44 years of age, the interaction effect of age on time was statistically significant across the other age groups indicating that the change in femicide over time is significantly different for girls and women 0–14, 45–59 and 60+ years of age compared with women 15–29 years of age.

## Discussion

We estimated femicide rates across 343 cities in nine Latin American countries and investigated differences by age and over time. The median city-level femicide rate across all cities was 3.9 per 100 000 females, well above the global rate of 1.1 per 100 000 female population.^13^ We found great variability between and within countries. Contrary to our hypothesis, the variability between countries was higher than the variability within countries, nonetheless, we found substantial variability within countries. Additionally, we found that women 15–29 and 30–44 years of age had the highest rates of femicide, consistent with our hypothesis that young adult women would be most affected. Finally, while our findings suggest that on average, for every decade, femicide rates may be slightly decreasing, we could not rule out the null hypothesis of no changes in femicide rates over time indicating the persistence of the problem. Moreover, these trends varied by country, with femicide rates potentially increasing in Mexico. We also found heterogeneity in trends over time by age indicating that the potential decline is not consistent across age groups. While our results indicate a decrease among the youngest and oldest women, we did not find statistically significant changes over time for women 15–44 years of age.

Prior research on violence in Latin America highlights important between country variability with lower homicide rates reported in Argentina, Chile and Costa Rica and higher homicide rates reported in Brazil, Mexico, Colombia and El Salvador.^4^ We find similar differences between countries when looking exclusively at femicides. Studies looking at spatial differences within country are either not disaggregated by gender or sex^24^ or tend to focus on larger geographical units within a single country.^16 22^ Research in Brazil between 2003 and 2007, for example, found that higher femicide rates were concentrated in states in the north and northeast.^16^ Our study finds that at the city level, between 2015 and 2019, femicides followed a similar pattern. While higher femicide rates are concentrated in many cities along the northeastern coast, other cities in Brazil report low femicide rates.

Overall, although there was greater variability between countries (67%), variability within countries was large (33%) suggesting that reporting country levels or even subnational femicide rates may obscure the variation between urban communities in the same country. One reason we might see greater variability between countries is because the type of violence leading to femicides may be influenced by macro level factors including patriarchal gender norms which may vary more between countries. Gender is a type of social stratification, and it may be that variations in this stratification system between countries are what leads to the observed variations in femicides between countries.^45^ The large variability observed between cities may instead be driven by local social policies (eg, education, employment, etc), policy response (eg, response to alerts and violence complaints), infrastructure (eg, transportation, panic buttons, etc) and local city culture (eg, social behaviour and accepted stereotypes) which may enhance or limit women’s access to prerequisites for safety and health. In a recent study, using data for 2010–2016, researchers found substantial variability in femicide rates (41%) within cities^31^ suggesting that there may be a spatial within-city components influencing femicides.

Our finding that overall femicide rates were generally highest among women 15–29 years of age is in line with previous estimates.^20^ One possible explanation for this finding may be the high levels of intimate partner violence during adolescence which may place young women at increased risk for femicide.^46^ Femicide, unlike male homicide, is largely committed by intimate partners^47^ and young women may have less options and thus be less able to leave violent and abusive relationships.^46^ However, our results also showed some important differences across countries. While most countries exhibited highest rates among women 15–29 years of age, Panama and El Salvador had higher median city femicide rates in women 30–44 years of age. This contradicts the notion that exposure to violence reduces with age.^48^ Future studies exploring femicides should explore factors that may explain these age differences across countries. There may be country-specific and city-specific policies that drive these differences including implementation of laws meant to protect girls and women such as child marriage laws.^49^

We also found that over time rates of femicide may be exhibiting a small decline, but we could not rule out the null hypothesis of no change. We find that trends vary by country, and that particularly in Mexico, femicides may be increasing. Homicide studies suggest that population ageing may place a downward pressure on overall homicide rates,^48^ but that this downward pressure may be attenuated by larger social problems. In Mexico, increases in femicide have been hypothesised to stem from increases in organised crime linked to drug trafficking and the militarisation that resulted to combat these problems.^48^ However, other social problems including a series of neoliberal policies which proceeded the War on Drugs including The North American Free Trade Agreement (NAFTA) and the maquila model may be worth exploring.^50^ These policy decisions, imposed regulations on regional production, leading to the employment of women, often from indigenous backgrounds and lower socioeconomic strata, in work environments that exposed them to violence. Ultimately, these measures may have contributed to an escalation in gender-related conflicts, exemplified by the well-known case of Ciudad Juarez in Mexico.^50^ Similar arguments that emphasise the gendered effects of fiscal policies can be made in other countries in the region. In Brazil, for example, recent austerity measures driven by policies that promote capitalistic gain over the protection of citizens have led to increases in gender inequality and violence against women.^51^ In Colombia, economic hardships and the lack of institutional social protections coupled with longstanding problems with armed groups have been linked to increases in femicides.^52^

Finally, our finding that the femicide rate among those 15–44 years of age does not show statistically significant changes over time is consistent with recent research in the region. A recent study in Brazil found that the rates of femicide for women 15–49 years of age have remained stable when comparing 1990 to 2019.^22^ The lack of progress in femicide prevention within this age group warrants further investigation and serves as a compelling call to action for Latin American governments.

This study had several limitations. First, we have no additional information to further characterise the murder of girls and women. The importance of characterising the different types of femicides for prevention purposes has been previously discussed.^53^ Yet, a recent United Nations report found that for every 10 reported cases of female homicide, four do not have information on perpetrator relationship.^13^ Advocating for and funding better data collection initiatives can help in identifying and specifically addressing different types of femicide. Moreover, while many countries in the region have now passed femicide legislation to protect girls and women, these crimes still go unpunished in many countries in the region where impunity rates are as high as 90%.^54 55^ Ensuring proper investigation of gender-based violence including femicides is only part of the solution. In addition, giving girls and women ways to report violence and protecting them once they do is critical. In Guatemala, for example, 40% of femicide cases had previously reported violence to authorities.^56^

Second, we have used vital registration homicide data to proxy femicide. At least two criticisms are expected. On the one hand, scholars have rightly argued that administrative data reflect femicide ‘thin counts’ meaning they represent only the surface manifestation of gendered violence and miss deaths resulting from the slow violence that women face every day under patriarchy, and which may lead to early death.^36^ Instead, centering patriarchal structures when counting femicides might entail including domestic violence and other abuse that may shorten the lives of girls and women^57^ or it might include, as initially argued by Lagarde when she coined the term feminicide,^58^ mortality due to state negligence. This understanding of femicide would mean that the analysis presented here reflects an undercounting of the true manifestation of lethal violence against girls and women. On the other hand, criticism of this proxy may stem from the lack of specificity in our operationalisation of femicide. The challenges in identifying gender-related motives have been widely explored and some proposals have been offered.^3 38^ For some, including all female homicides as femicides dilutes the theoretical and political impact of the term ‘femicide’, which was as originally coined by Russel to draw attention to a specific form of homicide.^39^ Yet, across time and geography, the characteristics of what is included continues to evolve making room for context-specific articulations of the problem.^59^

Another limitation related to the use of vital registration data is that vital statistics data varies in depth, quality, accuracy and completeness of the mortuary assessment across countries.^3860^,^62^ A recent assessment of vital statistics systems in these countries suggests that the strength of civil registration and vital statistics systems has varied with time,^61^ with Peru (not included in this study) and Colombia showing higher levels of under-reporting.^32^ According to other research, El Salvador is included among the countries in Latin America with poorer performing systems.^62^ In 2017, nearly 30% of deaths reported in El Salvador were ill-defined.^62^ Previous SALURBAL analysis has found that while missing values for key variables (age, sex, location and cause of death) were low among countries included in this study, nearly 20% of deaths in El Salvador were ill-defined.^34^ However, most of these ill-defined deaths in El Salvador are R99 codes, which should not include injuries (for which there is an analogous code, Y34), our main outcome in this study.^34^ While ill-defined codes have been redistributed as described previously,^34^ future research should explore other methods of redistribution. Other researchers have noted that deaths classified as accidents, suicide and undetermined intent are particularly relevant because they may include deaths that are femicides but were wrongly classified.^38^

Finally, death certificates often provide data on sex which is different from gender and thus renders invisible trans and gender non-binary individuals. In Argentina, starting in 2012, trans women could be officially registered as female,^63^ so some trans women will be included in the femicide rate after 2012. In other countries, the exclusion of trans women and girls would lead to an undercounting of femicides. Moreover, transfemicides is a social issue with its own specificities, in addition to the commonalities shared with the murder of cisgender women and should be further studied. Central and South America report over three-quarters of the killings of transgender and gender-diverse people in the world.^64^ Advocating for and funding initiatives trying to collect more gender sensitive data including civil society organisations and government efforts is necessary.

Despite these limitations, this is one of the first studies to explore trends in femicide in a large sample of Latin American cities and countries over the last two decades. Although much femicide research has demonstrated variations across countries, our study is among the first multinational studies to investigate city differences in femicide by age and across time. Using multilevel models, we were able to describe heterogeneity in femicide rates between countries and cities highlighting the need for further research that explores the relationship between features of the urban environment and femicide rates. This focus on cities builds on prior research that has found higher femicide rates in urban environments and is key to the development of local urban policies to reduce femicides. Results of this study add to our understanding of femicide-related mortality among girls and women in urban areas of Latin America, allowing for an understanding of temporal trends and enabling the development of hypotheses for this evolution. While it is known that many individual factors (eg, race, education and age) and city-level socioeconomic characteristics (eg, segregation, sanitation, etc) are associated with femicide risk, more studies are needed to examine the larger social structures that contribute to femicide.

## supplementary material

10.1136/bmjph-2024-000985 online supplemental file 1

## Data Availability

Data may be obtained from a third party and are not publicly available.
