# Supplementary material for: Characterising the killing of girls and women in urban settings in Latin America, 2000–2019: an analysis of variability and time trends using mortality data from vital registration systems
Source: BMJ Public Health. 2024 Jul 24;2(1):e000985. doi: 10.1136/bmjph-2024-000985 (PMC11812913; doi:10.1136/bmjph-2024-000985)

Table A. List of country with summary of data availability

			Years		City level age-adjusted femicide rate				
**Country**	**# cities**	**Total Years**	First	Last	Mean	Min	Max	Median	SD
**Argentina**	33	15	2005	2019	1.7	0	7.7	1.5	1.3
**Brazil**	152	20	2000	2019	4.5	0	27.3	4.0	3.0
**Chile**	21	20	2000	2019	1.0	0	6.3	0.9	1.1
**Colombia**	35	20	2000	2019	8.6	0	72.3	6.3	7.7
**Costa Rica**	1	20	2000	2019	2.1	1.3	3.0	2.1	0.5
**El Salvador**	3	19	2001	2019	12.6	3.9	23.5	12.0	5.5
**Guatemala**	3	11	2009	2019	11.3	0.5	24.0	12.2	6.5
**Mexico**	92	20	2000	2019	4.3	0	60.7	2.9	5.0
**Panama**	3	18	2002	2019	4.7	0	15.9	3.6	3.4

Table B. Number and proportion of cities and city-years reporting zero femicides overall and by country. Country specific percentage is calculated using country specific city count and city-years as the denominator.

[1]These are city-years where there were zero femicides during the time period 2000-2019

	Pooled analysis (2015-2019) (n=343 cities)	Longitudinal Analysis (2000-2019) (n=6,659 city-years)[1]
**Overall**	**1 (0.3%)**	**473 (7.1%)**
**Argentina**	**0**	**72 (14.5%)**
**Brazil**	**0**	**141 (4.6%)**
**Chile**	**1 (4.8%)**	**137 (32.6%)**
**Colombia**	**0**	**8 (1.1%)**
**Costa Rica**	**0**	**0**
**El Salvador**	**0**	**0**
**Guatemala**	**0**	**0**
**Mexico**	**0**	**113 (6.1%)**
**Panama**	**0**	**2 (3.7%)**

Equation A: Longitudinal model by level

$Femicide_{ijk}$ is measurement at time i on city j in country k

Level 1: Repeat measurement-level (Years) T_{ijk} is year(scaled to decade) for measurement i on city j in country k

$$Log(Femicide_{ijk}) = b_{0jk} + b_{1jk}T_{ijk} + b_{2jk}Age0\text{–}14 + b_{3jk}\text{Age30–44} + b_{4jk}\text{Age45–59} + b_{5jk}\text{Age60}^{+} + \log(pop_{ijk})$$

Level 2: City-Level

$$b_{0jk} = \gamma_{00k} + U_{0jk} \qquad U_{0jk} \sim N(0, \tau_{b00})$$
$$b_{1jk} = \gamma_{10k} + U_{1jk} \qquad U_{1jk} \sim N(0, \tau_{b10}) \qquad \text{Cov}(U_{0jk}, U_{1jk}) = 0$$
$$b_{2jk} = \gamma_{20k}$$
$$b_{3jk} = \gamma_{30k}$$
$$b_{4jk} = \gamma_{40k}$$
$$b_{5jk} = \gamma_{50k}$$

Level 3: Country-level

$$\gamma_{00k} = \alpha_{000} + U_{00k} \qquad U_{00k} \sim N(0, \tau_{\gamma 00})$$
$$\gamma_{10k} = \alpha_{100} + U_{10k} \qquad U_{10k} \sim N(0, \tau_{\gamma 10}) \qquad \text{Cov}(U_{00k}, U_{10k}) = 0$$
$$\gamma_{20k} = \alpha_{200}$$
$$\gamma_{30k} = \alpha_{300}$$
$$\gamma_{40k} = \alpha_{400}$$
$$\gamma_{50k} = \alpha_{500}$$

Full model:

$$\begin{aligned} Log(Femicide_{ijk}) = {} & \alpha_{000} + U_{00k} + U_{0jk} + \\ & ((\alpha_{100}) + (U_{10k}) + (U_{1jk}))T_{ijk} + \\ & \alpha_{200}(Age0\text{–}14) + \alpha_{300}(\text{Age30–44}) + \alpha_{400}(\text{Age45–59}) + \alpha_{500}(\text{Age60}^{+}) + \log\,(pop_{ijk}) \end{aligned}$$

Figure A. Country specific femicide rate in 2000 and country specific time trend, estimated as the random intercept and slope respectively, from a mixed model.

AR = Argentina, BR = Brazil, CL = Chile, CO = Colombia, CR= Costa Rica, GT= Guatemala, MX = Mexico, PA = Panama, SV = El Salvador.

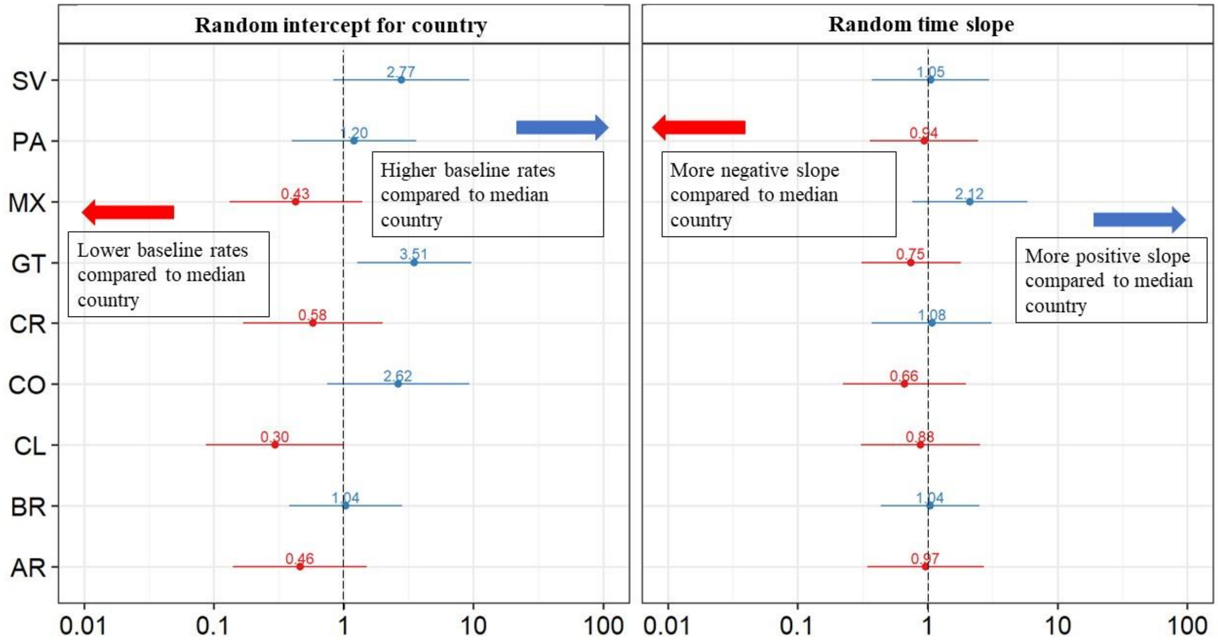

Supplement: online supplemental file 1 [file bmjph-2-1-s001.pdf]
